# Targeting of crosstalk between tumor and tumor microenvironment by β‐D mannuronic acid (M2000) in murine breast cancer model

**DOI:** 10.1002/cam4.1013

**Published:** 2017-02-17

**Authors:** Fatemeh Hosseini, Hadi Hassannia, Ahmad Mahdian‐Shakib, Farhad Jadidi‐Niaragh, Seyed Ehsan Enderami, Mohammadjavad Fattahi, Ali Anissian, Abbas Mirshafiey, Parviz Kokhaei

**Affiliations:** ^1^ Cancer Research Center and Department of ImmunologySemnan University of Medical SciencesSemnanIran; ^2^ Department of ImmunologySchool of Public HealthTehran University of Medical SciencesTehranIran; ^3^ Drug Applied Research CenterTabriz University of Medical SciencesTabrizIran; ^4^ Department of ImmunologyFaculty of MedicineTabriz University of Medical SciencesTabrizIran; ^5^ Department of Molecular MedicineZanjan University of Medical SciencesZanjanIran; ^6^ Shiraz institute for cancer researchShiraz, University of Medical SciencesShirazIran; ^7^ Department of Veterinary MedicineIslamic Azad UniversityAbharAbhar branchIran; ^8^ Department of Oncology‐PathologyImmune and Gene Therapy LaboratoryCancer Centre KarolinskaKarolinska InstitutetStockholmSweden

**Keywords:** β‐D‐mannuronic acid, M2000, breast cancer, metastasis

## Abstract

Metastasis is the main cause of death in breast cancer patients. Inflammatory processes following crosstalk between tumor cells and tumor microenvironment play an important role in progression and metastasis of cancer. Hence, targeting of these interactions may represent a novel promising strategy for breast cancer therapy. So, we investigated the effects of β‐D mannuronic acid (BDM), a new antiinflammatory agent, on 4T1 breast cancer cell line both in vitro and in vivo. Proliferation assays revealed low‐cytotoxic effect of BDM on 4T1 cells. However, BDM reduced activity of MMP‐2, MMP‐9 and significantly decreased the adhesion of 4T1 cells to extracellular matrix (ECM) in a dose‐dependent manner. The in vivo results demonstrated that BDM strongly inhibits tumor growth and increases lifespan as compared with control mice. The decrease in tumor mass was associated with decreased metastasis, recruitment, and frequency of inflammatory cells in tumor tissue. Our preclinical findings demonstrated that BDM therapy not only prevents formation of chronic inflammatory response but also inhibits crosstalk between tumor cells and their microenvironment, which is associated with reduction of tumor growth and metastasis arrest. Our data imply the use of BDM therapy in future clinical trials to open a new horizon for breast cancer therapy.

## Introduction

Breast cancer is the most common malignancy in women, accounting for 25% of all cancers, with nearly 1.7 million cases identified annually 1. The high mortality in breast cancer, 52,2000 cases per year, is largely due to the metastatic spread of tumor cells into vital organs 2. Surgery, chemotherapy, and targeted therapy are currently the main therapeutic options for breast cancer therapy. However, these approaches are not completely effective in metastatic breast cancer and are associated with high toxicities, unpleasant side effects, and tumor relapse 3, 4.

Tumor microenvironment plays an important role in cancer development and metastasis 5, 6. In malignant mammary tissue with metastatic properties, tumor cells recruit and instruct tumor‐infiltrating leukocytes (TILs) to favor tumor progression 7. Multiple interactions (“Crosstalk”) between tumor cells and TILs by secretion of cytokines, chemokines, and/or direct cell–cell contact, induce chronic inflammation and initiate premetastatic niche formation 8, 9. Tumor‐associated macrophages (TAMs), neutrophils, myeloid‐derived suppressor cells (MDSC), and regulatory T (Treg) cells are the major types of the TILs in the tumor microenvironment during chronic inflammation 6, 10, 11, which not only attenuate antitumor immune responses, but also they promote tumor growth, angiogenesis, lymphangiogenesis, and metastasis 12, 13, 14. The best evidence for the importance of inflammation in tumor progression comes from effectiveness of long‐term and regular use of nonsteroidal antiinflammatory drugs (NSAIDs), such as ibuprofen and aspirin, in prevention and treatment of colorectal, breast, and other cancers 15, 16. However, a long‐term use of NSAIDs for therapeutic purposes is associated with gastrointestinal, cardiovascular, and renal toxicities which limit their application 17. Thus, it is worthwhile to investigate for alternative antiinflammatory drugs that are safe for long‐term use.

Our previously patented novel antiinflammatory agent, (M2000, patented DE‐10247073), natural glycans especially β‐D mannuronic acid, is an attractive drug. The β‐D mannuronic acid (BDM) extracted from alginic acid sodium salt is a novel antiinflammatory agent with no evidence of toxicity in our preclinical toxicology study 18. Our previous studies have shown therapeutic efficacy of BDM with the high tolerability in various animal models such as experimental autoimmune encephalomyelitis 19, adjuvant‐induced arthritis 20, nephrotic syndrome 21, and acute glomerulonephritis 22. Currently, we are analyzing the efficacy of BDM in clinical trials in patients with ankylosing spondylitis (IRCT: 2013062213739N1) and rheumatoid arthritis (IRCT: 2014011213739N2).

Our previous findings showed that BDM is a potent matrix metalloproteinase (MMP) inhibitor, especially the gelatinases (MMP‐2 and MMP‐9), that play important roles in tumor invasion, metastasis, and angiogenesis 23. All these clues led us to test the antitumor and antimetastatic effects of BDM in 4T1 breast cancer model, which is animal model for stage IV human breast cancer in immune competent BALB/c mice. Regarding the high metastatic capacity and inflammatory microenvironment of 4T1 tumor model, this model can better mimic the clinical situation compared to a mouse model bearing human tumor xenografts 24, 25. In this study, we investigated the antiinflammatory and anticancer properties of BDM on 4T1 murine breast cancer cells.

## Methods

### Cell line and experimental design

**Figure 1:**
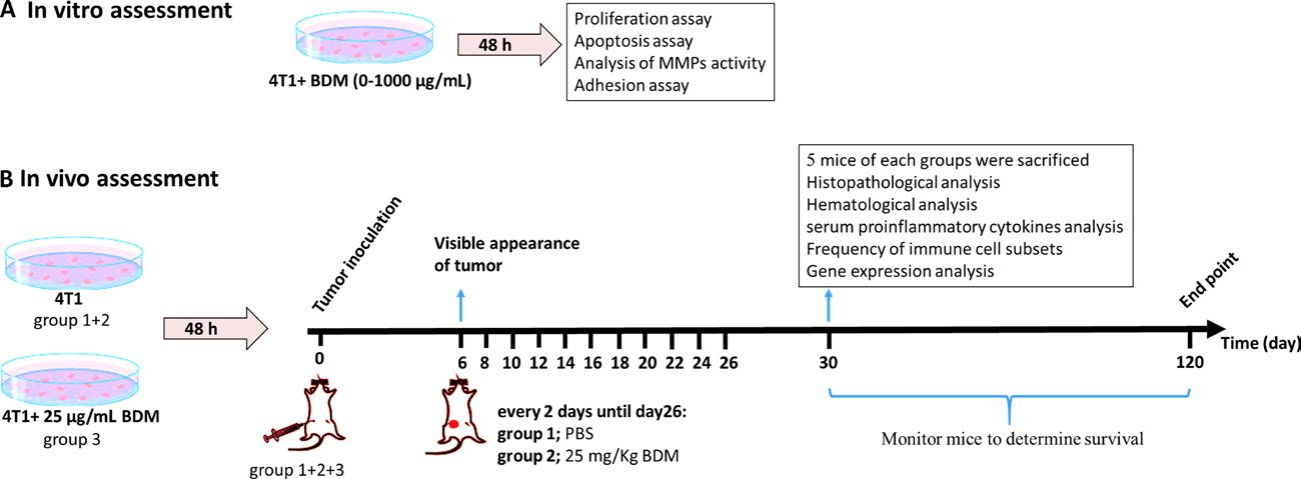
Experimental design scheme. (A) Schematic illustration of the invitro assessment (B) Schematic illustration of the in vivo assessment, animals were divided into 3 groups: treated with PBS (group 1); treated with 25 mg/kg of BDM after tumor induction (group 2); cell line pre‐treated with 25 µg/ml of BDM before inoculation (group 3). Tumor growth was measured every 2 days.

The 4T1 murine BALB/c breast cancer cell line was obtained from the National Cell Bank of Iran (Pasteur Institute of Iran, Tehran, Iran). Cells were maintained in RPMI 1640 medium supplemented with 10% heat‐inactivated fetal bovine serum, 2 mmol/L L‐glutamine, and penicillin/streptomycin (100 units/mL), at 37°C in 5% CO2 humidified atmosphere. Cell lines were routinely tested for mycoplasma contamination. BDM was extracted from alginic acid sodium salt (Sigma‐ Aldrich, St. Louis, MO) by acid hydrolysis method as described previously 18 and then dissolved in PBS (5 mg/mL) as a stock solution. This study was designed to test the antitumor effect of BDM on 4T1 cell line both in vitro and in vivo (experimental design is shown in Fig. 1).

### In vitro assessment of BDM effect on 4T1 cells

#### Cell proliferation

To test the effects of BDM on cell proliferation, 4T1 cells were plated overnight in 96‐well plates at a density of 5 × 10^3^ cells/well. The medium was replaced with the same media containing different concentrations of BDM (0, 5, 25, 250, 500, 1000 *μ*g/mL). Plates were incubated at 37°C in 5% CO2 for 48 h and then the cells were pulsed with 1 *μ*C_i_ of [3H]‐thymidine (PerkinElmer, Waltham, MA, USA) for 18 h. The cells were harvested after 18 h of incubation and transferred to scintillation fluid for measurement of radioactive thymidine incorporation by a beta counter (Wallac 1410 Liquid Scintillation Counter, Pharmacia, Sweden). All cultures were performed in triplicate and repeated at least twice.

#### Apoptosis assay

To evaluate the cell apoptosis, 4T1 cells were treated for 48 hours with different concentrations of BDM (0, 25, 250, 500, 1000 *μ*g/mL). Cells were washed twice with PBS and 1 × 10^5^ cells were resuspended in 100 *μ*L binding buffer, then incubated with 5 *μ*L of Annexin V‐FITC and propidium iodide (PI) as a counterstain for 15 min at room temperature in the dark, according to the manufacturer's instruction (BD Biosciences, San Diego, CA, USA). Finally, at least 10^4^ cells per sample were analyzed with a FACSCalibur flow cytometer (Becton Dickinson) and data were analyzed using Flowjo software (version 7).

#### Gelatin zymography

To test MMP activity, 4T1 cells were treated for 48 hours with different concentrations of BDM (0, 25, 250, 500, 1000 *μ*g/mL) in a serum‐free medium. The supernatants were then centrifuged at 400 *g* for 5 min at 4°C, and the total protein of the supernatant was normalized with BCA protein assay kit. Equal amounts of protein (4 *μ*g) were electrophoresed under nonreducing conditions in to 8% SDS‐PAGE gel copolymerized with gelatin (1 mg/mL, Sigma‐Aldrich) as previously described 26. The gels were incubated overnight at 37°C in substrate buffer containing 50 mmol/L Tris‐HCl, pH 8.0, 5 mmol/L CaCl2, 0.02% NaN3, and stained with Coomassie blue. The dried gel was scanned and the densitometric measurements of the bands were calculated using the UN‐SCAN‐IT gel digitizing software (Silk Scientific, Inc. Orem, UT).

#### ECM cell adhesion assay

Cell adhesion assay was performed as described previously with a slight modification 27. Briefly, 4T1 cells were pretreated with different concentrations of BDM (0–1000 *μ*g/mL) for 48 h, and then trypsinized. Cells (5 × 10^5^ cells/well) were added into 96‐well plates which were precoated with collagen I, fibronectin, and laminin (Sigma). Plates were incubated for 1 h at 37°C, then nonadherent cells were removed by a gentle washing four times with PBS, and the remaining cells were stained with 0.1% crystal violet for 5 min. After washing, the precipitates were dissolved by addition of 30% acetic acid, and the absorption was obtained at 590 nm. The percentage of inhibition was expressed using control wells as 100%.

### In vivo antitumor study

#### Murine model of metastatic breast cancer

After acclimatization, 27 BALB/c mice (6‐ to 8‐week‐old female, inbred, weight=18–20 g) were randomly divided into three groups (nine mice for each group) (Fig. 1B): (1) control (treated with PBS), (2) therapeutic model (treated with 25 mg/kg of BDM after tumor induction), and (3) cell line pretreated model (cell line treated with 25 *μ*g/mL of BDM before tumor inoculation). The dose of BDM was assigned as the effective and safest dosage based on in vitro and pilot study. Tumors were induced by injecting 4T1 cells (5 × 10^5^ cells in total volume of 0.1 mL PBS) into the mammary fat pad. PBS and BDM were administered intraperitoneally in the opposite flank of groups 1 and 2, respectively. All experiments involving mice were approved by animal ethics committee of Tehran university of medical sciences.

#### Tumor growth and survival study

The tumor sizes were measured every 2 days using digital calipers and volumes were calculated using the following formula: V = length × (width)^2^/2. At day 30 posttumor inoculation, five mice of each groups were killed (Fig. 1B). The tumors, selected internal organs (lungs, spleen, liver, kidneys, heart, brain, and tumor regional lymph nodes) and blood samples were collected for histology, flow cytometry, gene expression, hematological, and serum analyses. The survival was investigated in the remaining mice.

#### Histological analysis

For histological evaluation, all dissected organs were fixed in 10% buffered formalin and embedded in paraffin using standard methods. Three 4‐*μ*m serial sections were taken from each tissue and stained with hematoxylin and eosin (H&E). All sections were scanned using a digital slide scanner (Pannoramic DESK, 3D Histech, Hungary). The incidence of metastases to various organs were investigated in mice. Percentage of lung metastatic area to total lung area was also analyzed with the Panoramic Viewer software.

#### Tissue preparation and analysis of MDSC and Treg cells

Spleens, tumors, and lymph nodes were gently dissociated under 40‐*μ*m mesh cell strainer (BD Biosciences) for single‐cell isolation. After red blood cell lysis (Sigma‐Aldrich), single cells were washed and resuspended in PBS. These cells were labeled with fluorescence‐conjugated antibodies against mouse Gr1‐APC and CD11b‐PE (BD, PharMingen) to identify MDSC, or with PE‐conjugated CD4 and FITC‐conjugated FOXP3 to identify Treg cells and isotype‐matched controls. For intracellular staining, cells were fixed and permeabilized with Foxp3 staining buffer before incubation with the conjugated antibody or isotype control. Samples were analyzed with a FACSCalibur flow cytometer (Becton Dickinson) and data were analyzed using Flowjo software (version 7).

#### Hematological and cytokines analysis

Total white blood cells were counted on a Neubauer chamber. Smears stained with Giemsa solution were investigated for the presence of the granulocyte, lymphocyte, and monocyte populations. The serum samples were used to measuring IL‐1β, IL‐6, and TNF‐α cytokines by commercially available ELISA kits (R&D, USA), according to the manufacturer's instructions.

#### Gene expression analysis by real‐time PCR

We measured the gene expression of major chemokines that are involved in the recruitment of TILs into tumor site including S100A8, S100A9 (chemotactic for MDSCs), CCL22 (chemotactic for Tregs), and CCL2, CCL9 (chemotactic for monocytes and neutrophils) 28, 29. We also measured the expression of major cytokines and factors that promote angiogenesis and metastasis, including TGFβ, VEGF, MMP‐2, MMP‐9, and HIF‐1α 9. Total RNA was extracted from tumor tissues using GeneAll RiboEx kit (GeneAll, Korea) and subsequently converted into cDNA by high‐capacity cDNA reverse transcription kit (Applied Biosystems, Foster City, CA, USA). The β‐actin was used as a reference control. The PCR assay was performed by StepOne Real‐time PCR System (Applied Biosystems) using the SYBR Green fluorescence quantification system (Takara). Each primer blasted for gene specificity and validated using a standard curve across the dynamic range of interest with a single melting peak. The relative mRNA expression levels were calculated using the 2−^ΔΔCT^ method.

### Statistical analysis

All experiments were performed three times and the data are presented as mean ± SEM. Depending on the type of experiment, data were tested using two‐tailed Student's *t*‐test, log‐rank test, Pearson correlation coefficient, or one‐way ANOVA with post hoc Bonferroni correction. All statistics and graphs were made in Graphpad Prism (v5.03). *P < 0.05, **P < 0.01, ***P < 0.001, and ****P < 0.0001 were considered statistically significant.

## Result

### Reduced activity of MMPs and adhesion of 4T1 cell to ECM by BDM

**Figure 2:**
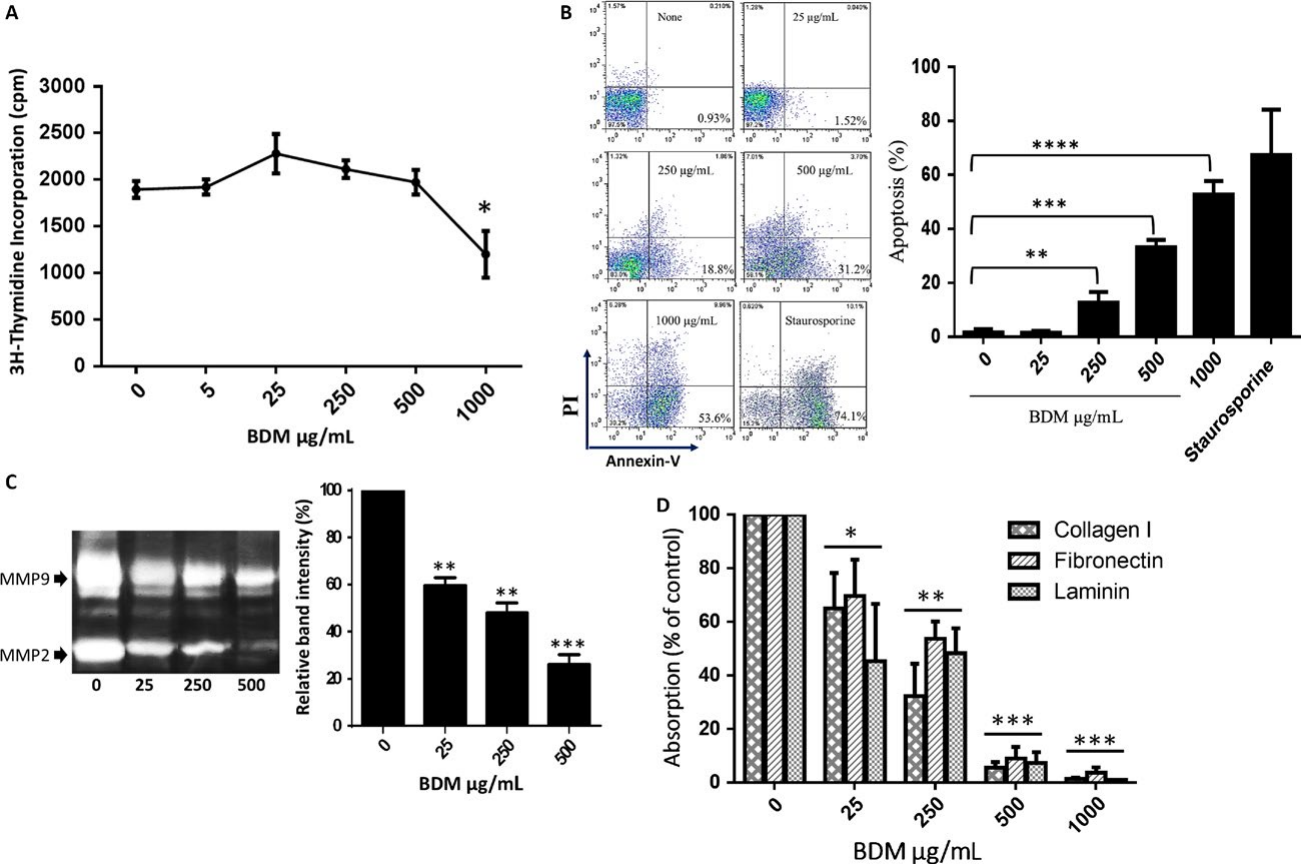
Treatment of 4T1 cells with different concentrations of BDM. (A) Effect of BDM on proliferation of 4T1 cells. The proliferation assay was performed by 3H‐thymidine incorporation assay. (B) The percentage of apoptotic cells was determined by Annexin V‐FITC/PI staining. (C) The effects of BDM on MMP2 and MMP9 activity. (D) Effect of BDM on 4T1 cells adhesion to collagen I, fibronectin and laminin.

To find safest and effective dosage, we treated cultured 4T1 cells with various concentrations (0, 25, 125, 250, 500, and 1000 *μ*g/mL) of BDM for 48 h and then measured proliferation, apoptosis, MMP activity, and ability of cells to adhere extracellular matrix (ECM). BDM did not effectively reduce the in vitro cell proliferation of 4T1 cells except dose 1000 (Fig. 2A). This result is in agreement with our previous findings; we showed that high tolerability of WEHI‐164 as a sensitive cell line against increasing quantities of BDM compared with steroidal and nonsteroidal drugs tested 23. However, BDM increased the apoptosis rates of 4T1 cells in a dose‐dependent manner. The representative histograms of flow cytometry showed the apoptosis rates of 4T1 cells to be 1.23%, 1.52%, 12%, 33%, and 52% when they underwent 48 hours incubation with BDM at concentrations 0,25, 250, 500, and 1000, respectively (Fig. 2B). Also, ~ 40–70% activity of MMP‐2 and MMP‐9 in 4T1 cells were decreased in response to treatment with BDM in a dose‐dependent manner (Fig. 2C). Our ECM adhesion assay data revealed that BDM could significantly reduce the adhesion of 4T1 cells to collagen I, fibronectin, and laminin which are the major components of the ECM (Fig. 2D). Based on these results, we selected dose 25 μg/ml (low concentrations, no toxicity, significantly reduce the MMP activity and adhesion of 4T1) as a suitable dose for cell line pretreated model in in vivo study.

### BDM inhibits tumor growth, prevents metastasis, and prolongs survival in tumor‐bearing mice

**Figure 3:**
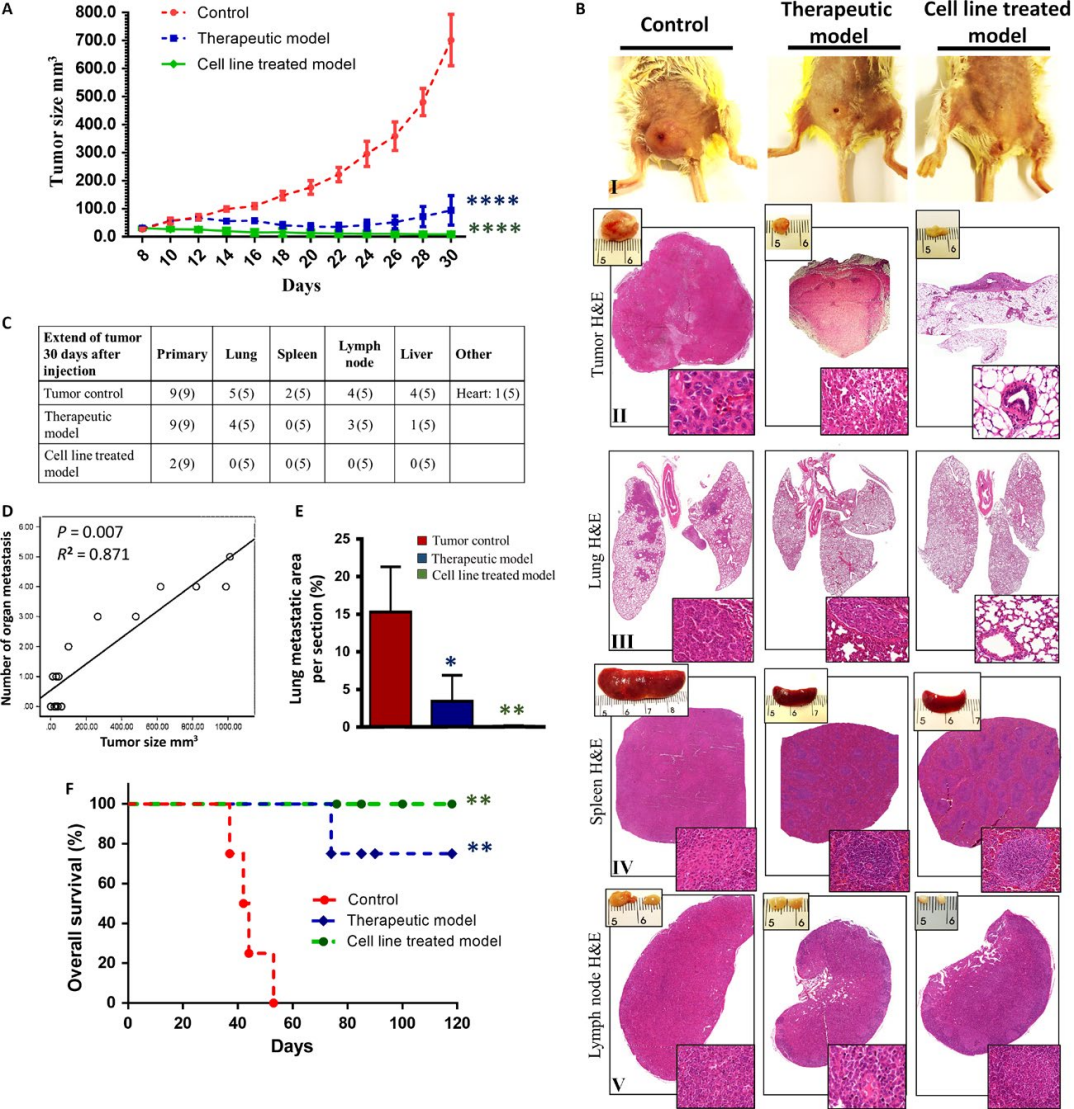
Effects of BDM on tumor growth and metastasis in a 4T1 tumor bearing mice model. (A) Growth curves of 4T1 tumors treated by PBS, and the BDM treated groups. (BI) Three representative mice from each group were shown. (BII‐V) Bright field imaging and H&E staining of the tumor tissue, lung, spleen, and lymph node on day 30 after tumor inoculation. The areas boxed (Magnification: 40×). (C) Incidence of metastasis to different organs (number of animals with metastasis), nine mice were used in each group, 5 mice on day 30 after tumor inoculation sacrificed and 4 mice for survival assay. (D) Association of number of organ metastasis with tumor size in 15 samples. (E) Lung metastases were quantified as percentage of metastatic area per lung section. (F) Kaplan–Meier survival curves of mice treated with BDM or PBS control during 120 days’ post inoculation.

Following assessment of the in vitro antitumor effect of BDM on 4T1 cells, we further determined the in vivo antitumor effect of BDM in murine breast cancer model. Mice were divided in to three groups including control, therapeutic model, and cell line treated (*n* = 9). Since it was supposed to investigate the tumorigenic ability of 4T1‐treated cells in mice, we need to select safest dose of BDM. Therefore, we selected dose 25 *μ*g/mL (low concentrations, no toxicity, significantly reduce the MMP activity and adhesion of 4T1) as a suitable dose for cell line pretreated model in in vivo study. The schematic study design is shown in Figure 1B. Primary tumor appearance was observed on day 6 in all animals. At day 30 after implantation, there was a significant decrease in tumor size in BDM‐treated groups (Fig. 3A). As compared to the control mice, the tumor volume was 86.5% and 98.7% smaller in group 2 and 3, respectively (****P < 0.0001).

To evaluate whether BDM therapy inhibits the ability of 4T1 tumorigenicity in the murine breast cancer, H&E‐stained sections were analyzed (Fig. 3B_II‐V_). Histologically, in the control group, the morphology of tumor cells in primary tumors were composed of high levels of mitosis (averaging 5–7 mitosis/ high‐power fields [hpf]), neutrophilic inflammation, and high necrosis area in center of the tumor which was presumably due to hypoxia 30. Although in therapeutic group, the mitotic rate was low (1 mitosis/hpf), small areas of neutrophilic inflammation and necrosis area in edge of tumors were observed. Moreover, in cell line‐treated group, the mitotic rate was very low (>1 mitosis/5 hpf) and no visible infiltrated inflammatory cells and necrosis area were observed. As shown in Figure  3B_IV, V_, spleen and lymph node in control mice had dramatically higher size compared to the BDM‐treated groups. As shown, the splenomegaly and lymphomegaly were accompanied by diminished white pulp and B‐cell follicles, respectively.

Moreover, BDM therapy inhibited the metastatic ability of 4T1 (Fig. 3C). All of mice in control group had metastases, the incidence of metastasis in the lungs (100%), spleen (40%), lymph node (80%), liver (80%), heart (20%), and no metastases in the kidneys and brain. The incidence of metastasis in therapeutic group were found in the lungs (80%), spleen (0%), lymph node (60%), liver (20%), and heart (20%). As shown (Fig. 3A), there is a visible reduction in tumor size in group 2, which occurred 8 days after the initiation of BDM therapy. This period, 14 days after tumor cell inoculation, seems that provide the required time for 4T1 tumor cells spreads through the blood or lymphatic vessels. Surprisingly, there is no metastasis to other organs in cell line‐treated group (0%). We also observed significant correlation between the tumor size and the number of organ metastasis (*R* = 0.871, **P = 0.007) (Fig. 3D). Similarly, the decreased dissemination of tumor cells was associated with reduction of lung metastasis area in BDM‐treated groups compared with control group (Fig. 3E). Lung metastasis area in therapeutic group 77.8 % (*P = 0.014) and cell line‐treated group 98.5% (**P = 0.002) were fewer than control group. In parallel with inhibition of tumor growth, mice treated with BDM had a prolonged lifespan (Fig. 3F). Mean survival time in control group was only 44 ± 3.3 days, while in BDM‐treated groups, survival time was >120 days (**P = 0.0067).

### BDM therapy reduces inflammation and immunosuppressive cells in tumor‐bearing mice

**Figure 4:**
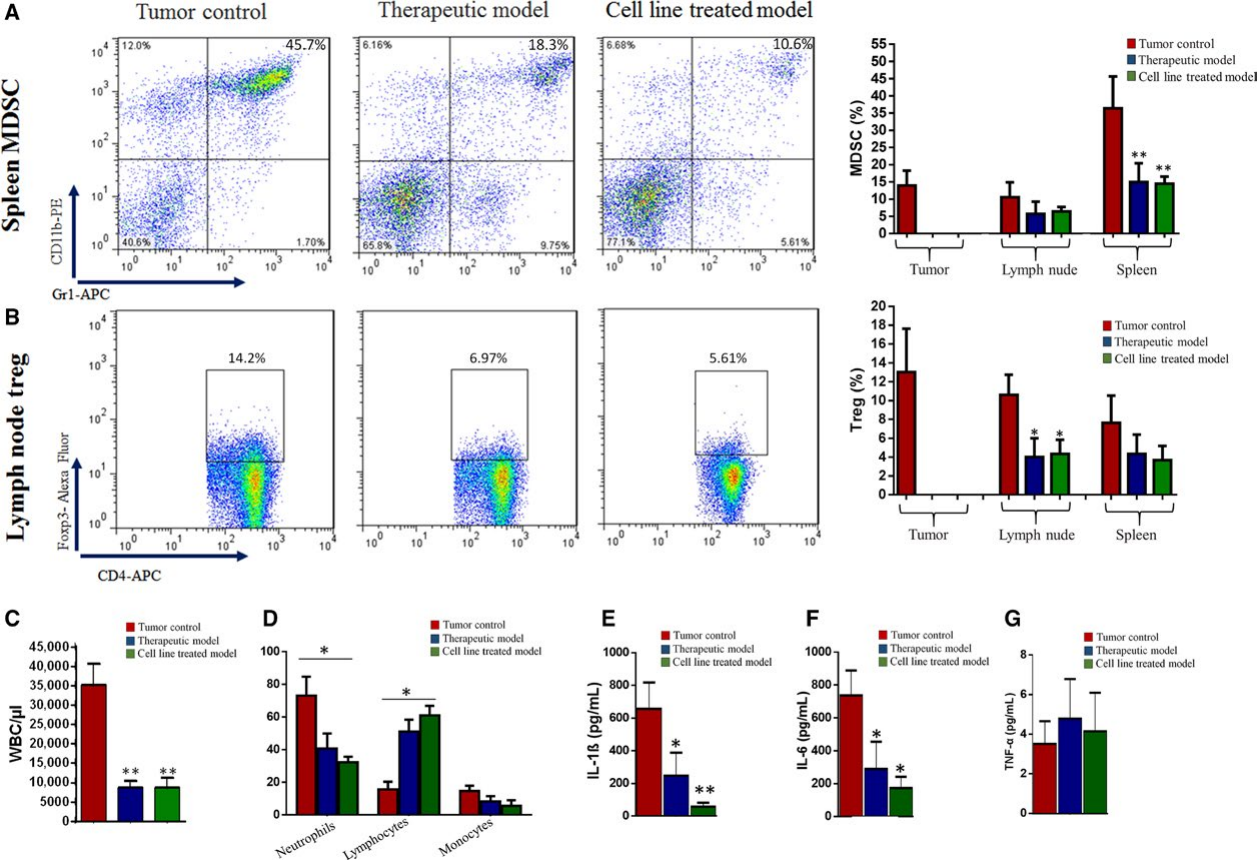
Cytometry analysis of leukocytes isolated from tumor, spleen, lymph node, blood and levels of serum pro‐inflammatory cytokines. (A) FACS analysis of spleen MDSCs from each group is shown (left panel). Double‐positive Gr‐1+CD11b+ cells from the tumors, spleen, lymph node are shown (right panel). MDSCs significantly reduced in the spleens of BDM treated groups. (B) FACS analysis of spleen Treg from each group is shown (left panel). Double‐positive CD4+FOXP3+ cells from the tumors, spleen, lymph node are shown (right panel). Treg significantly reduced in the lymph node of BDM treated groups. (C), (D) Complete blood counts from each group. (E), (F), (H) The levels of serum IL‐1β, IL‐6 and TNF‐α from each group. Results are presented in picograms of cytokine per ml of serum (pg/ml). (n =5 mice/group). * Because of the small size of the tumor in BDM treatment groups, we were unable to determine the frequency of these cells in tumor tissue.

We determined the frequencies of MDSC and Treg in tumors, spleens, and lymph nodes by flow cytometric analysis at 30 days after tumor induction. Representative dot plots demonstrating the method used for analysis the percent of MDSC (Fig. 4A) and Treg (Fig. 4B) are shown. Due to small size of primary tumor in BDM treated groups, we were unable to determine the frequency of these cells in tumor tissue. BDM therapy significantly decreased MDSCs in spleen (Fig. 4A right panel) and significantly decreased Treg in lymph node (Fig. 4B right panel). On the other hand, there was no change in the frequency of MDSC in lymph node and frequency of Treg in spleen. Also, the frequencies of MDSC and Treg were directly correlated with tumor size and metastasis (data not shown). In order to evaluate the impact of BDM therapy on systemic inflammatory response in the murine breast cancer, we analyzed the WBC count, neutrophil, lymphocyte, monocyte ratio, and serum proinflammatory cytokines. The results showed that mean of WBC count in control group significantly increased compared with the BDM‐treated groups (Fig. 4C). Moreover, control group had higher neutrophil to lymphocyte ratio (favorable components of inflammatory process) than BDM‐treated groups (Fig. 4D). The increase in proinflammatory cells was also accompanied by increased serum levels of proinflammatory cytokines. Our results showed that IL‐1β (Fig. 4E) and IL‐6 (Fig. 4F) levels were significantly increased in control group compared with BDM‐treated groups, whereas TNF‐α showed no difference (Fig. 4G).

### BDM suppresses the expression of inflammatory chemokines and tumor‐promoting cytokines

**Figure 5:**
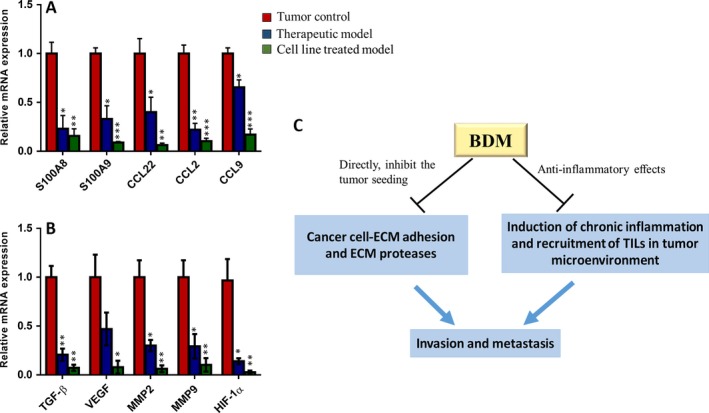
Effects of BDM on chemokines and cytokines expression in primary tumors. RT‐qPCR was used to quantify 10 genes expression in tumor isolated from each group, 30 days after tumor inoculation. Histograms represent: (A) The relative mRNA expression of major chemokines for recruitment of TILs to the tumor site. (B) The relative mRNA expression of major factors that promote angiogenesis and metastasis. (C) schematic hypothesis: dual role for BDM anticancer effects.

To better define the underlying mechanisms of the antitumor effects of BDM, we compared RNA expression profiles in tumors isolated from each group. Analyzing RT‐qPCR data by the comparative ΔΔC_t_ method indicated that in BDM‐treated groups, expressions of inflammatory chemokines (S100A8, S100A9, CCL22, CCL2, CCL9) substantially decreased compared with the control animals (Fig. 5A). As expected, we also observed tumor‐promoting factors (TGFβ, VEGF, MMP‐2, MMP‐9, and HIF‐1α) were reduced in BDM‐treated groups compared with the control animals (Fig. 5B). These data suggested that BDM inhibits influx of TILs into primary tumors site, which is concomitant with reduced tumor‐promoting factors.

## Discussion

One hundred years ago, Steven Paget proposed the “Seed and Soil” hypothesis, suggesting that a fertile “Soil” (the microenvironment) is essential for the anchoring of “Seed” (the tumor cells) to grow 31. There is accumulating evidence from clinical and preclinical studies that inflammatory responses following crosstalk between tumor cells and surrounding microenvironment are essential for development of tumor 5. Hence, factors that regulate tumor interaction are attractive target for therapeutic applications 32. We have shown that BDM has antiinflammatory, high safety, and potential inhibitory effects against MMP‐2, MMP‐9 18, 23, 33. In this study, we investigated the potential antitumor and antimetastatic effects of BDM on 4T1 breast cancer cell line both in vitro and in vivo. The tumor invasion and metastasis require the ability of tumor cells to degrade and adhere to ECM 34. Our in vitro results showed that BDM significantly reduces the MMP‐2, MMP‐9 activities (involved in degradation of the ECM) and adhesion of 4T1 cells to collagen I, fibronectin, and laminin (major components of the ECM) in a concentration‐dependent manner. Several studies report that β1 integrin families mediate principle cell surface adhesion receptor of ECM 35 and interact with MMP activity in melanoma, glioma, and breast cancer cells 36, 37, 38. In invasive tumors, MMPs and integrins are expressed in high levels in tumor cells suggesting that there is possible interaction between the two protein families, however, studies attempted to identify the role of MMPs in cancer cell adhesion. In addition, we proposed that BDM similar to other NSAIDs, has different antiinflammatory mechanisms such as L‐selectin shedding and inhibition of integrin activation and expression 39. However, further investigation on whether BDM regulates cell adhesion molecules is needed. The in vivo results demonstrated that BDM strongly inhibits tumor growth and increases lifespan as compared with control mice. Decrease in tumor mass was associated with decrease recruitment of inflammatory cells to tumor microenvironment and prevents tumor metastasis. Furthermore, significant reduction of MDSCs, Treg and neutrophil both in frequency and chemotactic factor have been observed in BDM‐treated groups. Our study adds to the repertoire of a number of independent studies that have demonstrated the critical role of multiple interactions (“Crosstalk”) between tumor cells and their microenvironment in tumor development and metastasis. We found that in vitro treated 4T1 cell line with 25 *μ*g/mL BDM significantly reduced the MMP‐2, MMP‐9 activities and adhesion of 4T1 cells to collagen I, fibronectin, and laminin which inhibited 77.7 % tumor incidence after inoculation without any effect in viability of cell line (Fig. 3C). Decrease in tumor incidence, size, and metastasis in group 3 may be associated with inhibition of tumor seeding in tumor implantation site. Previous studies have shown that the main biophysical problem associated with solid tumor therapy is poor penetrating capabilities of some drugs (e.g., antibody), which is in part related to a high packing density of the constituent cells and acidic microenvironment of tumor cells, leading to low efficacy and/or inhibits drugs uptake, such as doxorubicin and mitoxantrone 40, 41, 42. It seems that BDM with a low molecular size (200 Dalton) and mannuronic acid structure may able to overcome these obstacles. Although the BDM molecular mechanism is not clearly elucidated, based on the mannosyl structure of BDM, predicts its binds to mannose receptor 43 family, without activating them. MRs are essential components in cancer cell adhesion and cell traffic within the lymphatic system and blood circulation 44. Also, the expression of high mannose glycoproteins in cancer tissues may play important role in the regulation of tumor genesis processes including cancer cell adhesion, migration, and signaling 45. As proof of concept, several mannose‐based cancer therapy approaches have been reported. For instance, recent observations indicate that blocking of mannose ligand residues on the endothelial cell surface by metaperiodate treatment significantly reduced tumor cell adhesion and metastasis 46. Moreover, adherence of tumor cells to extracellular matrix was dramatically abolished by soluble mannose‐binding lectin treatment 47. It has been supported by antitumorigenic effects of mannose‐recognizing plant lectins 48, use of mannosylated chitosan for prostate cancer immunotherapy 49, application of mannosylated radiotracers for solid tumor staging 50, and positive correlation between levels of high mannosylation glycoproteins and the tumor burden in both mouse and human during breast cancer progression 51. It was also demonstrated that the mannosylated proteins stimulate immune system with higher immunogenicity compared to peptides or proteins that are not mannosylated 52, 53. Our data approved that development of primary tumors and metastasis is enhanced by the complex network of inflammatory chemokines/cytokine that regulate TILs recruitment 54. Therefore, it seems that decrease in tumor growth and metastasis in BDM‐treated groups may be associated with inhibition of tumor cell crosstalk with surrounding and induction of chronic inflammation.

In summary, our study provides the first evidence of therapeutic effects of BDM (M2000) in breast cancer model and raises the hypothesis that antiinflammatory agents can be effective in cancer therapy. Our results (Fig. 5C) suggest a dual role for BDM anticancer effects (antiinflammatory effects and directly, inhibit the tumor seeding), however, this claim needs further investigations in other cancers. Regarding the high safety of BDM, it is worthwhile to design a clinical trial for investigating novel BDM therapy in human solid tumors (particularly, invasive breast cancer) and preventive approach in higher risk women with a family history of breast cancer.

## Conflicts of Interest

The authors declare no financial or commercial conflict of interest.
